# Can the intermittent low-speed function of left ventricular assist device prevent aortic insufficiency?

**DOI:** 10.1007/s10047-020-01234-4

**Published:** 2021-01-09

**Authors:** Hiroki Kohno, Goro Matsumiya, Yoshiki Sawa, Norihide Fukushima, Yoshikatsu Saiki, Akira Shiose, Minoru Ono

**Affiliations:** 1 Department of Cardiovascular Surgery, Chiba University Graduate School of Medicine, 1-8-1 Inohana, Chuo-ku, Chiba, 260-8677 Japan; 2 Department of Cardiovascular Surgery, Osaka University Graduate School of Medicine, Osaka, Japan; 3 Department of Transplantation, National Cerebral and Cardiovascular Center, Osaka, Japan; 4 Division of Cardiovascular Surgery, Tohoku University Graduate School of Medicine, Sendai, Japan; 5 Department of Cardiovascular Surgery, Faculty of Medical Sciences, Kyushu University, Fukuoka, Japan; 6 Department of Cardiac Surgery, The University of Tokyo, Tokyo, Japan

**Keywords:** Continuous-flow left ventricular assist device, Intermittent low-speed function, Aortic insufficiency

## Abstract

Aortic insufficiency (AI) is known to associate with a persistently closed aortic valve during continuous-flow ventricular assist device support. Some devices carry an intermittent low-speed (ILS) function, which facilitates aortic valve opening, but whether this function prevents AI is unknown. In this study, the Jarvik 2000 device, which is programmed to reduce the pump speed each minute for 8 s, was chosen to examine this potential effect. Prospectively collected data of 85 heart transplant-eligible Jarvik 2000 recipients who met the study criteria (no pre-existing AI and aortic valve surgery) were retrospectively analyzed for the incidence, correlating factors, and clinical outcomes of de novo AI. All data were provided by the Japanese Registry for Mechanically Assisted Circulatory Support. De novo AI occurred in 58 patients, 23 of whom developed at least moderate AI during a median support duration of 23.5 months. Freedom from moderate or greater AI was 84.4%, 66.1% and 60.2% at 1, 2 and 3 years, respectively. Multivariate analyses revealed that progressive AI was correlated with decreased pulse pressure after implantation (hazard ratio 1.060, 95% confidence interval 1.001–1.127, *p* = 0.045). No correlation was found for mortality or other adverse events, including stroke, bleeding, infection, pump failure, hemolysis, and readmission. The benefit of the Jarvik 2000′s current ILS mode against AI appears to be minimal. However, in this limited cohort where all recipients underwent implantation as a bridge to transplantation, the impact of de novo progressive AI on other clinical adversities was also minimal.

**Supplementary Information:**

The online version contains supplementary material available at 10.1007/s10047-020-01234-4.

## Introduction

Despite the increasing number of patients on continuous-flow left ventricular assist device (cf-LVAD) support, certain complications associated with the device remain unresolved. One such complication is aortic insufficiency (AI). Over the course of time, the continuous, high turbulent pressure generated by these devices on the aortic root and valve may induce pathological changes, such as root dilatation, valve degeneration, and commissural fusion, and contribute to the development and progression of AI [1].

Progressive AI after cf-LVAD implantation can have serious consequences. It creates a continuous retrograde flow loop that limits effective forward flow, thus increasing the risk of end-organ malperfusion and right heart failure and potentially affecting long-term survival [1].

With accumulating evidence that AI occurs more frequently in patients with a persistently closed aortic valve during cf-LVAD support [1–10], strategies to prevent AI have now focused on the optimization of the pump speed to promote aortic valve opening [1, 2, 4–15]. In some cf-LVADs, including the HeartWare HVAD (HeartWare, Inc., Framingham, MA, USA), the HeartMate III (Abbott Laboratories, Abbott Park, IL, USA), and the Jarvik 2000 (Jarvik Heart Inc., New York, NY, USA), the devices are equipped with a function that facilitates intermittent aortic valve opening by periodically minimizing pump flow. This function thus offers the prospect of preventing AI progression; however, there is only a paucity of studies that have addressed this potential benefit [13–15].

The Jarvik 2000 is an axial cf-LVAD that contains a miniaturized intraventricular pump and an intermittent low-speed (ILS) function, which reduces pump flow for 8 s every minute. While other devices with a similar ILS function have only just recently entered the Japanese market, the Jarvik 2000 was approved for commercial use in late 2013 and has since been used increasingly in the country. This has provided us a valuable opportunity to examine the ILS effect against AI. In this study, we aimed to determine the incidence of de novo AI in patients on the Jarvik 2000, demonstrate the beneficial effect of ILS against AI in the light of published data, predict who would be susceptible to AI development despite the ILS effect, and evaluate the outcomes of those who developed AI during follow-up.

## Materials and methods

The present study was approved by the ethics review board of the Japanese Registry for Mechanically Assisted Circulatory Support (J-MACS). J-MACS is a government-funded national registry aimed at improving treatment outcomes and safety measures for left ventricular assist device (LVAD) patients [16]. In Japan, all LVAD implantations are done strictly as a bridge to transplantation. Between January 2014 and January 2019, 172 patients from 22 centers were registered and received the latest cone-bearing version of the Jarvik 2000, which was connected to an ILS controller. This controller follows a programmed algorithm that slows the pump speed to 6000 rpm (the normal operating range is 8000–12,000 rpm) for 8 s every minute. All data used in this study were provided by J-MACS, which contains a database populated by collecting pre-specified information at designated intervals, or on an occurrence basis, from the participating hospitals of the registry (Appendix 1). The data provided for this study were pre-adjudicated by independent organizations and de-identified to the study investigators. A written informed consent that allowed prospective collection, analyses, and conditional disclosure of information was obtained from all patients.

The prospectively collected data of the 172 recipients of the Jarvik 2000 were reviewed. The recipients with pre-existing AI (57 patients), or unknown aortic valve status (23 patients), or who underwent aortic valve surgery before or at the time of Jarvik 2000 implantation (12 patients) were excluded from the study cohort. From this cohort, 6 patients, who remained alive during the study period but lacked post-implantation data of the aortic valve, were also excluded. After the exclusion process, 85 patients remained. The data of these 85 patients were analyzed for the incidence, correlating factors, and clinical outcomes of AI after implantation.

AI severity was categorized by J-MACS as follows: none; trivial or mild; moderate; or severe (including moderate-to-severe). To obtain a clear-cut picture of the effects of ILS on AI development, only the patients who were reported as no AI before implantation were included in the study cohort. In the subsequent analyses, the data of the Jarvik-supported patients with at least moderate AI were compared with those with less than moderate AI.

### Statistical analysis

Values are summarized as frequencies and proportions for categorical data, mean ± standard deviation for parametrically distributed continuous data, and median and interquartile range (IQR) for non-parametrically distributed continuous data. A Cox proportional hazard model was used to identify predictors and potential correlates of AI. The variables included in multivariate analyses were those that showed a significant correlation on univariate analyses. The rate of survival and freedom from first event were estimated using the Kaplan–Meier method, and the log-rank test was used to compare time-to-event outcomes between groups. The patients were censored for heart transplantation, removal of the LVAD, and death. A *p* value of < 0.05 was considered statistically significant.

## Results

The median support duration of the 85 Jarvik LVAD recipients was 715 days (IQR 313–1076; maximum 1505 days). Twelve recipients underwent heart transplantation, 2 underwent LVAD explantation after recovery of the left ventricle, 1 had the LVAD converted to an extracorporeal biventricular support system because of hemolysis and right heart failure, and 58 were receiving ongoing support during the study period.

### De novo AI

**Fig. 1:**
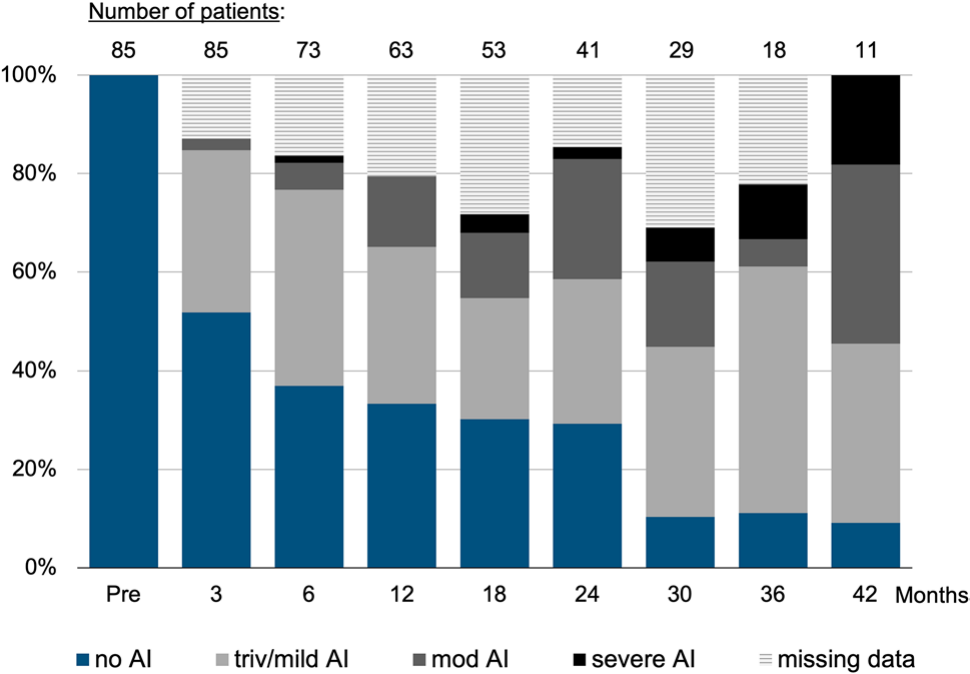
Aortic insufficiency (AI) status with time after implantation. Each color represents one of the following: no AI; trivial or mild AI; moderate AI; severe AI; or unavailable/missing data

**Fig. 2:**
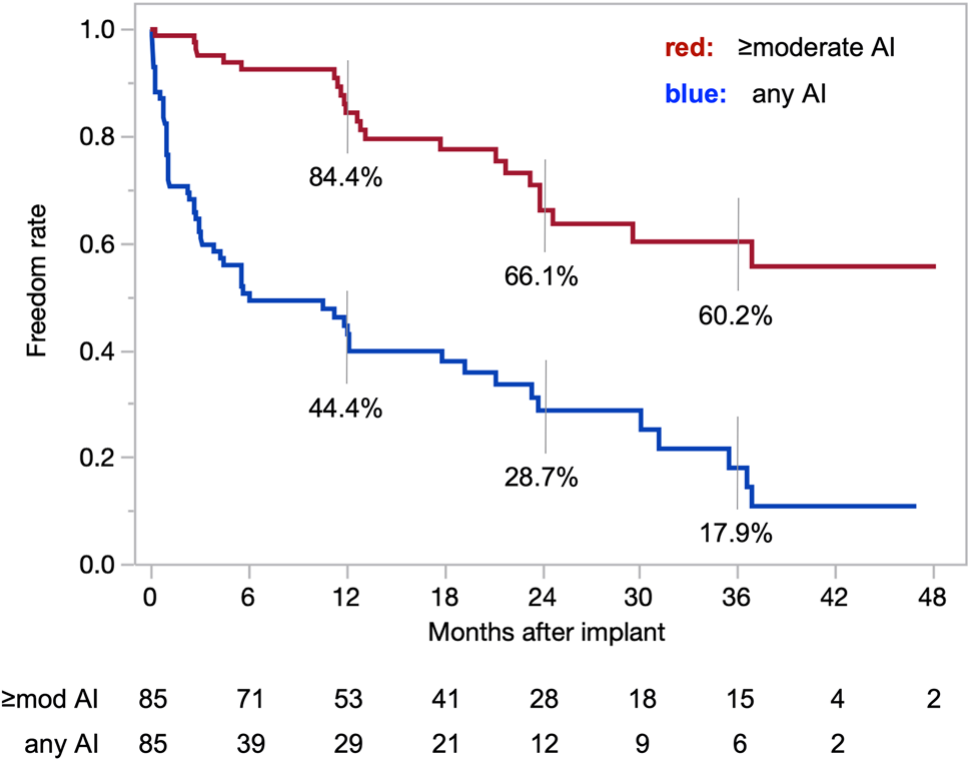
Freedom from aortic insufficiency (AI) after implantation

De novo AI occurred in 58 (68.2%) patients. There were 23 (27.1%) patients who progressed to moderate or severe AI within 49 months of implantation. The distribution of AI severity with time after implantation is shown in Fig. 1. At 1, 2 and 3 years, actuarial freedom from moderate or greater de novo AI was 84.4%, 66.1% and 60.2%, respectively (Fig. 2).

### Comparison of baseline characteristics

Table 1 lists the pre-implantation baseline variables that were used to determine the risk factors for developing moderate or greater AI after implantation. The patients who developed at least moderate AI were older, smaller in size, and had lower cardiac index than those who did not develop moderate or greater AI.

**Table 1:** Baseline characteristics

Variable	< Moderate AI	≥ Moderate AI	HR	95% CI	*p*
	(*n* = 62)	(*n* = 23)			
Age (years)	40 (28–46)	44 (35–60)	1.04	1.01–1.08	0.008
Body surface area (m^2^)	1.5 ± 0.2	1.4 ± 0.2	0.09	0.01–0.87	0.037
Female	30 (48.4%)	15 (65.2%)	1.45	0.64–3.61	0.390
INTERMACS level 1	3 (4.8%)	1 (4.4%)	0.73	0.04–3.48	0.748
INTERMACS level 2–3	56 (90.3%)	22 (95.7%)	2.55	0.53–45.6	0.290
Cause of heart failure					
Dilated cardiomyopathy	35 (56.5%)	15 (65.2%)	1.04	0.45–2.59	0.935
Ischemic heart disease	8 (12.9%)	0	1.8e^−9^	1.49–1.48	0.106
Hypertrophic cardiomyopathy	6 (9.7%)	1 (4.4%)	0.59	0.03–2.57	0.507
Others	5 (8.1%)	6 (26.1%)	2.52	0.90–6.21	0.077
Previous cardiac surgery	25 (40.3%)	10 (43.5%)	1.03	0.44–2.35	0.937
On ECMO/VAD support	18 (29.0%)	2 (8.7%)	0.29	0.05–0.99	0.095
Echocardiographic data					
Mitral regurgitation ≥ moderate	26 (42.6%)	14 (60.9%)	1.67	0.73–4.00	0.226
Tricuspid regurgitation ≥ moderate	17 (28.3%)	9 (40.9%)	1.70	0.70–3.96	0.223
Left ventricular end-diastolic diameter (cm)	6.3 ± 1.3	6.4 ± 1.5	1.05	0.74–1.50	0.788
Left ventricular end-systolic diameter (cm)	5.7 ± 1.3	5.8 ± 1.5	1.06	0.75–1.54	0.762
Left ventricular ejection fraction < 0.3	52 (83.9%)	18 (78.3%)	0.89	0.35–2.70	0.820
Left ventricular ejection fraction < 0.2	37 (59.7%)	13 (56.5%)	0.99	0.43–2.31	0.975
Pressure studies (mm Hg)					
Pulmonary capillary wedge pressure	18.4 ± 9.9	17.9 ± 7.5	0.99	0.94–1.03	0.576
Mean pulmonary artery pressure	25.3 ± 9.9	24.2 ± 9.9	0.99	0.93–1.02	0.366
Central venous pressure	8 (5–13)	8 (5–13)	0.99	0.91–1.05	0.786
Cardiac Index (L/min/m^2^)	2.3 (1.9–2.8)	1.9 (1.7–2.3)	0.40	0.16–0.92	0.030
Pre-implantation blood data:					
Creatinine (mg/dL)	0.9 (0.6–1.1)	0.8 (0.6–1.0)	0.56	0.17–1.58	0.286
Total bilirubin (mg/dL)	0.8 (0.6–1.3)	0.7 (0.5–1.0)	0.76	0.32–1.47	0.450
Albumin (g/dL)	3.7 ± 0.5	3.8 ± 0.4	0.99	0.47–2.08	0.984
Platelet count (× 10^3^)	203 (165–279)	175 (142–227)	0.96	0.91–1.01	0.170

Values are presented as number (%) of patients for categorical variables, mean ± standard deviation for parametrically distributed continuous variables, and median (interquartile range) for non-parametrically distributed continuous variables

*AI* aortic insufficiency, *ECMO* extracorporeal membrane oxygenation, *VAD* ventricular assist device, *HR* hazard ratio, *CI* confidence interval

### Comparison of operative and postoperative parameters

All implantations were done using cardiopulmonary bypass. Two implantations were performed with cardiac arrest. At implantation, 43 patients required at least one concomitant procedure, which included mitral valve replacement/repair, tricuspid valve repair, atrial septal defect closure, maze surgery, previous LVAD removal, and omentopexy. All patients had the outflow graft anastomosed to the ascending aorta.

The operative and postoperative variables are shown in Table 2. The time required for surgery and the procedures performed were similar between groups. During the postoperative follow-up period, the patients who developed at least moderate AI had faster heart rates, lower pulse pressures, lower hemoglobin levels, and lower platelet counts.

**Table 2:** Intraoperative and post-implantation data

Variable	< Moderate AI	≥ Moderate AI	HR	95% CI	*p*
	(*n* = 62)	(*n* = 23)			
Operation time (min)	460 (365–553)	451 (376–561)	0.99	0.99–1.00	0.584
Cardiopulmonary bypass time (min)	120 (101–170)	110 (98–158)	0.99	0.99–1.01	0.717
Aortic cross-clamp	1 (1.6%)	1 (4.4%)	5.40	0.29–27.9	0.195
Concomitant surgery	33 (53.2%)	10 (43.5%)	0.71	0.30–1.61	0.412
Mitral valve surgery	7 (11.3%)	1 (4.4%)	0.32	0.02–1.53	0.183
Tricuspid valve surgery	23 (37.1%)	7 (30.4%)	0.82	0.32–1.93	0.664
Post-implantation vital parameters*:					
Heart rate (beats/min)	81 (75–89)	82 (77–91)	1.04	1.00–1.08	0.040
Mean blood pressure (mm Hg)	74.0 ± 6.6	72.2 ± 9.8	0.97	0.92–1.03	0.301
Pulse pressure (mm Hg)	19 (12–24)	8 (4–19)	0.91	0.87–0.96	< 0.001
Post-implantation blood data*					
Hemoglobin (g/dL)	11.1 ± 1.3	10.6 ± 1.0	0.63	0.42–0.95	0.027
Platelet count (× 10^3^)	179.1 ± 61.1	153.8 ± 47.3	0.89	0.81–0.97	0.010
Albumin (g/dL)	3.9 (3.5–4.0)	3.7 (3.4–3.9)	0.43	0.16–1.34	0.142
Lactate dehydrogenase (U/L)	534 (447–725)	518 (441–642)	1.00	0.99–1.01	0.997
Total bilirubin (mg/dL)	1.0 (0.7–1.9)	1.4 (0.9–1.6)	1.20	0.82–1.57	0.313
Creatinine (mg/dL)	0.7 (0.6–0.9)	0.7 (0.6–0.9)	0.67	0.13–2.49	0.592
Brain natriuretic peptide (pg/mL)	178 (95–274)	255 (147–349)	1.00	0.99–1.01	0.129
Prothrombin time (INR)	2.4 (2.1–2.5)	2.3 (2.0–2.5)	0.63	0.24–1.34	0.289

Values are presented as number (%) of patients for categorical variables, mean ± standard deviation for parametrically distributed continuous variables, and median (interquartile range) for non-parametrically distributed continuous variables

*AI* aortic insufficiency, *INR* international normalized ratio, *HR* hazard ratio, *CI* confidence interval

*Represent the average of all available data collected after implantation and before the development of greater than moderate AI

### Predictors of moderate or greater de novo AI

Table 3 shows the results of the multivariate analyses that compared the perioperative variables of the Jarvik recipients. The analyses identified an increased risk of moderate or greater AI development with reduced pulse pressures after implantation (hazard ratio 1.060; 95% confidence interval 1.001–1.127; *p* = 0.045).

**Table 3:** Independent risk factors of moderate or greater de novo aortic insufficiency

Variable*	Univariate	Multivariate		
	*p*	HR	95% CI	*p*
Age	0.008	1.026	0.987–1.071	0.193
Body surface area	0.037	0.045	0.001–1.545	0.088
Preoperative cardiac index	0.030	0.412	0.127–1.168	0.097
Postoperative average heart rate	0.040	1.026	0.968–1.083	0.372
Postoperative average pulse pressure	< 0.001	0.943	0.887–0.999	0.045
Postoperative average hemoglobin	0.027	1.074	0.552–2.226	0.839
Postoperative average platelet count	0.010	0.932	0.820–1.046	0.243

*HR* hazard ratio, *CI* confidence interval

*Only the variables that showed a significant correlation on univariate analyses are shown here

### Post-implantation outcomes

**Fig. 3:**
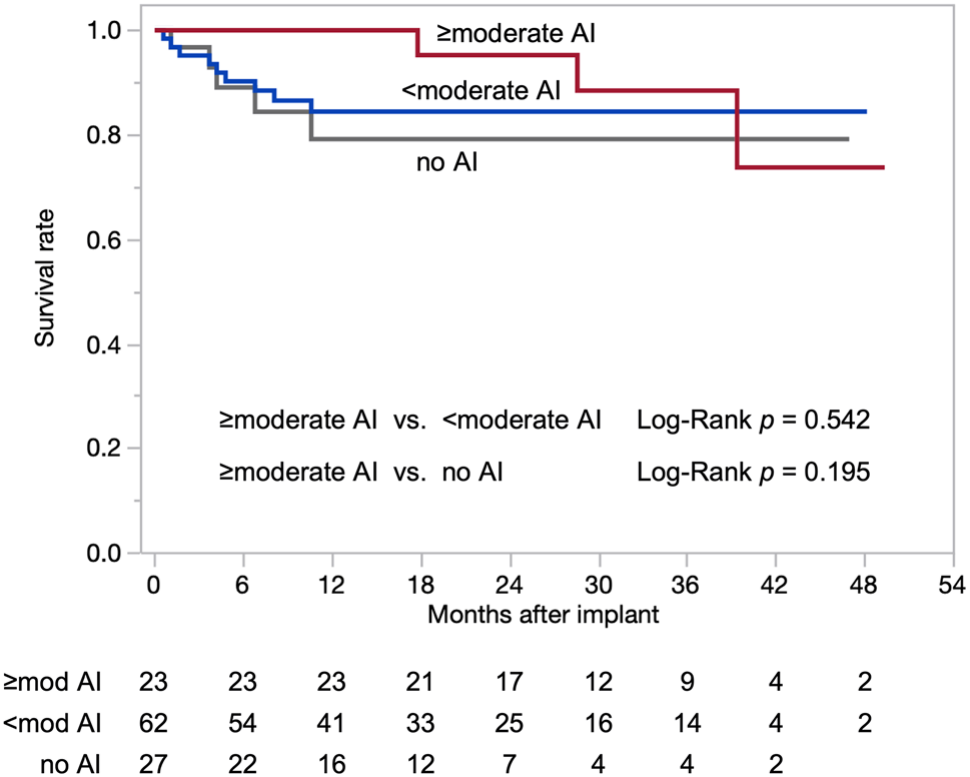
Survival after implantation based on aortic insufficiency (AI) severity

Twelve patients died while on the LVAD support, and the causes of death were stroke (6 patients), sepsis (3 patients), nonocclusive bowel ischemia (1 patient), and multiorgan failure preceded by an operator (patient) error-related device malfunction (2 patients; one experienced complete battery depletion and the other suffered from a pump shutdown due to an external component damage). One patient, who developed severe AI after 42.1 months of implantation, underwent LVAD explantation due to hemolysis and was converted to an extracorporeal biventricular support system because of concomitant right heart failure. However, no difference was observed in survival between patients who developed moderate or greater AI and those who did not (Fig. 3). We also found no direct association between moderate or greater AI with other adverse events, including bleeding, infection, stroke, pump failure, hemolysis, and readmission (Table 4).

**Table 4:** Impact of moderate or greater aortic insufficiency on post-implantation adverse outcomes

	Number of:			Correlation of ≥ mod AI with events by Cox analysis:		
	Patients affected	Events	Events accompanied by ≥ mod AI	HR*	95% CI	*p*
Major bleeding^a^	23	63	5	0.128	0.030–0.544	0.005
Gastrointestinal bleeding	6	19	3	0.143	0.018–1.125	0.065
New infection^b^	40	66	9	0.439	0.213–0.905	0.026
Device-related infection	21	34	2	0.841	0.196–3.611	0.815
Neurological dysfunction^c^	31	48	4	0.411	0.119–1.067	0.070
Stroke	28	43	4	0.430	0.147–1.261	0.124
Device malfunction^d^	25	44	11	0.242	0.110–0.530	< 0.001
Pump failure	7	11	6	0.082	0.009–0.728	0.025
Hemolysis^e^	6	8	3	0.153	0.017–1.378	0.094
Readmission (all-cause)	45	142	35	0.394	0.266–0.583	< 0.001
Death (all-cause)	12	12	3	0.665	0.178–2.484	0.544

*AI* aortic insufficiency, *HR* hazard ratio, *CI* confidence interval

^a^Significant blood loss resulting in death, reoperation, hospitalization or blood transfusion

^b^Any clinical signs of infection requiring anti-microbial and/or surgical treatment in an area that was healed or without previous infection

^c^Any new, temporary or permanent, focal or global neurologic deficit

^d^Failure of any component related to the device

^e^Any clinical signs of device-related hemolysis occurring after the first 72 h after implantation

*Note that a hazard ratio < 1 indicates a negative correlation between ≥ moderate AI and the event concerned

## Discussion

AI is a common sequela of cf-LVAD implantation with a reported frequency of up to 78% [3–14, 17, 18]. The progression of AI during cf-LVAD support may negatively impact the patients’ clinical outcomes; the aggravation of the continuous retrograde flow loop can cause severe restriction of forward blood circulation and induce malperfusion of the vital organs [1]. In view of minimizing these adverse effects, recent studies have suggested reducing the LVAD pump speed to promote aortic valve opening, as progressive AI has been consistently shown to associate with a closed aortic valve [1–15]. In this context, LVADs that are equipped with the ILS technology, which facilitates aortic valve opening, may have the potential to prevent AI. In this study, given the relatively high number of patients with the Jarvik 2000 LVAD in Japan, we chose this device to evaluate this effect. We found that 27.1% of the Jarvik recipients, who had no AI before implantation, developed at least moderate AI during a median support duration of 715 days; the incidence at 1 year was 15.6%. We initially hypothesized that these results would be better than those reported for other cf-LVADs without the ILS function.

A review of the literature [4–7, 11, 12] shows that the occurrence rate of moderate or greater AI in patients supported with other cf-LVADs ranges from 5 to 19% and the incidence at 1 year was from 5 to 28% (Appendix 2). Notably, these results represent those from previous studies that included cf-LVAD patients who were not subjected to the ILS effect, and, contrary to our expectations, the results were not significantly different from ours. Furthermore, the reports [13, 14] from European centers where the HeartWare HVAD is provided with a software that activates a programmed low-speed algorithm (the Lavare cycle), which produces an ILS-like effect, showed that the development of AI was rare in the patients supported with the HVAD. In those studies, moderate or severe AI developed in < 3% of the HVAD patients during a median support duration of approximately 400–600 days, and the 1-year incidence rate was < 2%. Interestingly, the same studies also revealed that AI progression was common if the aortic valve remained closed, or only occasionally opened, during support.

Despite the clear association between AI and aortic valve opening, not many data exist on the frequency of the valve opening in relation to AI development. The ILS mode of the Jarvik 2000 facilitates aortic valve opening only 13% of the time (8 s every minute) during LVAD support, and it is possible that this level of assistance may be insufficient to prevent AI. Previous studies [4, 12–14, 17] have shown that progressive AI is associated with not only a persistently closed aortic valve but also an intermittently opening aortic valve. Cowger et al. [4] defined intermittent opening as 1–2 openings in 3 systoles and found that progressive AI was significantly correlated with this frequency of aortic valve opening. The same authors [4] hypothesized that intermittently opening aortic valves, when subjected to continuous backflow pressure, may undergo pathological changes, including commissural fusion and disruption of the valve leaflets, similar to those seen in a closed aortic valve.

In this study, we discovered that low pulse pressure after implantation was a strong predictor of AI progression. Unfortunately, due to the lack of post-implantation data on the aortic valve opening status, we could not associate pulse pressure values with the frequency of aortic valve opening. Previous studies [2, 9], however, have shown that low pulse pressure measurements during LVAD support can indicate infrequent or no opening of the aortic valve. In a previous Jarvik 2000 study, Myers et al. [2] observed that a pulse pressure of < 15 mm Hg resulted in aortic valve opening in only 24% of the time, and a pulse pressure > 15 mm Hg was predictive of the valve opening 65% of the time. The median pulse pressure of our Jarvik recipients who developed moderate or greater AI was 8 mm Hg compared to 19 mm Hg in those who did not, thus indicating that the aortic valve may have rarely opened in the patients who progressed to moderate AI. Interestingly, in the present study, the same group that showed lower pulse pressure during support also had lower cardiac index before support (Table 1), implying the role of the native cardiac function as to whether it can generate a systolic gradient across the aortic valve after cf-LVAD implantation. We also suspect that the difference in the occurrence rate of AI between our study and other related studies [13, 14] may be due to the difference in, aside from the follow-up time, the proportion of patients with a persistently or semi-persistently closed aortic valve.

The etiology of AI development in cf-LVAD patients may be multifactorial, and the reported risk factors include advanced age, female gender, small body size, small left ventricular dimension, large aortic root diameter, position and angle of the outflow graft, short heart failure duration before support, high systolic blood pressure during support, and longer support duration [4–13, 15, 17, 19]. It can be anticipated that longer support duration may associate with AI progression, particularly if the patient already possesses a risk factor, such as old age, that can naturally cause AI to develop. In this study, we also found that smaller body size was, to some extent, correlated with AI development, although, despite several speculations [4, 8], the exact mechanism for their relationship remains unclear.

The clinical consequences of AI during cf-LVAD support have yet to be investigated. Notably, despite the foreseeable outcome of progressive AI in patients with cf-LVAD, the literature is not abundant on this topic, perhaps reflecting the scarcity of data on long-term cf-LVAD therapy. So far, the general conjecture that progressive AI, with its unique closed-circuit effect, reduces survival rate has not been proven [5, 7, 8, 12], and we similarly did not find this association in the present study. On the contrary, previous studies [9] have reported increased readmission rate in patients with AI; however, this increase was not found in our study, probably because of the small number of patients in the AI group. Furthermore, this study was not specifically designed to determine the fate of the LVAD patients with progressive AI, as all patients were waitlisted for transplantation (i.e., AI may have influenced the waiting time) and those with pre-existing AI were excluded from the analyses. In light of the increasing demand for long-term support, a larger study over a longer follow-up period is certainly warranted.

Several limitations exist in the present study, including the retrospective design, the small study population, and the lack of a comparative control population, as all patients were subjected to the ILS effect. In addition, our study lacks information on aortic valve opening status, which has consistently been shown to correlate with AI. Likewise, data on other suggested correlates, such as aortic root dimension, aortic valve morphology, outflow graft orientation, and pump speed, were missing in most or all of our patients. Regarding the pump speed, we can only assume that the patients were optimized individually as recommended by the current guidelines. Furthermore, our study was based on registry data that were self-reported; thus, unreported data were present and misreported data may have existed but cannot be adjusted for.

## Conclusion

The potentially protective role of ILS against AI in patients supported with the Jarvik 2000 appears questionable, as the incidence of AI was not different from that reported in the studies of other cf-LVADs without the ILS function. The patients who developed AI had markedly reduced pulse pressures after implantation, implying that the aortic valve may have rarely opened in those patients. These results suggest that the Jarvik 2000′s 8-s-every-minute ILS effect is insufficient to prevent AI, particularly if the aortic valve, for whatever reason, fails to open most of the time during support. Perhaps, if the ILS duration can be adjusted to individual patients, the results may differ. This study also showed that de novo progressive AI was not indicative of increased mortality or other adverse events during a median period of 2 years (and up to 4 years) after implantation in patients who are waiting for transplantation. However, in view of the growing number of patients requiring a longer support period, further evaluation is necessary.

## Supplementary Information

Below is the link to the electronic supplementary material.

Supplementary file1 (DOC 28 KB)

Supplementary file2 (DOC 58 KB)
