# Supplementary material for: Can the intermittent low-speed function of left ventricular assist device prevent aortic insufficiency?
Source: J Artif Organs. 2021 Jan 9;24(2):191–8. doi: 10.1007/s10047-020-01234-4 (PMC8154761; doi:10.1007/s10047-020-01234-4)
Supplement: Supplementary file 1 — Supplementary file1 (DOC 28 KB) [file 10047_2020_1234_MOESM1_ESM.doc]

**Appendix 1.** J-MACS participating hospitals (and hospital representatives) involved in this study (in alphabetical order):

Chiba University Hospital (Goro Matsumiya, MD, PhD), Dokkyo Medical University Hospital (Hirotugu Fukuda, MD, PhD), Ehime University Hospital (Hironori Izutani, MD, PhD), Gunma Cardiovascular Center (Masahiko Ezure, MD, PhD), Hokkaido University Hospital (Yoshiro Matsui, MD, PhD), Kyushu University Hospital (Akira Shiose, MD, PhD), National Cerebral and Cardiovascular Center (Norihide Fukushima, MD, PhD), Okinawa Prefectural Nanbu Medical Center & Children's Medical Center (Takafumi Miyara, MD, PhD), Osaka University Hospital (Yoshiki Sawa, MD, PhD), Saitama Medical University International Medical Center (Hiroyuki Nakajima, MD, PhD), The University of Tokyo Hospital (Minoru Ono, MD, PhD), Tohoku University Hospital (Yoshikatsu Saiki, MD, PhD), and Tokyo Women's Medical University Hospital (Hiroshi Niinami, MD, PhD).
