# Supplementary material for: Can the intermittent low-speed function of left ventricular assist device prevent aortic insufficiency?
Source: J Artif Organs. 2021 Jan 9;24(2):191–8. doi: 10.1007/s10047-020-01234-4 (PMC8154761; doi:10.1007/s10047-020-01234-4)
Supplement: Supplementary file 2 — Supplementary file2 (DOC 58 KB) [file 10047_2020_1234_MOESM2_ESM.doc]

**Appendix 2**. Previous studies on de novo AI development during cf-LVAD support

| Authors  (year) | Device  type (n) | Support duration  (days) | AI at baseline | De novo AI criteria | Development of de novo AI | Freedom from  de novo AI |
| --- | --- | --- | --- | --- | --- | --- |
| Pak et al. [3]  (2010) | HM II (63)  (XVE (67)) | (mean)  cf-LVAD: 257  pf-LVAD: 176 | no AI | ≥ mild-mod | cf-LVAD:  9/63 (14.3%)  pf-LVAD:  4/67 (6.0%) | at 6M, 12M:  cf-LVAD: 83.6%, 75.2%  pf-LVAD: 94.5%, 88.9% |
| Cowger et al. [4]  (2010) | HM II (53)  (XVE (25)) | (median)  cf-LVAD: 239  pf-LVAD: 169 | ≤ triv | ≥ mod | 15/78 (19.2%)  including pf-LVAD | at 6M, 12M, 18M:  cf-LVAD: 86%, 72%, 36%  pf-LVAD: 100%, 80%, 80% |
| Hatano et al. [17]  (2011) | EvaH (4)  DuraH (1)  Jarvik (3)  HM II (1)  (Nipro (28)) | (mean)  cf-LVAD: 765  pf-LVAD: 508 | ≤ trace | ≥ mild-mod | cf-LVAD:  7/9 (77.8%)  pf-LVAD:  2/28 (7.1%) | n/a |
| Soleimani et al. [18]  (2012) | HM II (55)  HW (8) | (mean) 315 | ≤ mild | ≥ mild-mod | 6/63 (9.5%) | at 6M, 1Y:  100%, 68.4% |
| Rajagopal et al. [11]  (2013) | HM II (139)  HW (6)  Ventra (3)  (XVE (33))  (Nova (3)) | (median)  cf-LVAD 371  pf-LVAD 84 | ≤ triv | ≥ mod | cf-LVAD:  19/148 (12.8%)  pf-LVAD:  2/36 (5.6%) | at 6M, 12M, 18M:  (estimates)  cf-LVAD: 94%, 91%, 89%  pf-LVAD: 98%, 84%, 84% |
| Aggarwal et al. [5]  (2013) | HM II (79) | (median) 761 | ≤ triv | ≥ mild | ≥ mild  41/79 (51.9%)  ≥ mod  4/79 (5.1%) | n/a |
| Jorde et al. [6]  (2014) | HM II + HW (174) | (mean) 344 | ≤ trace | ≥ mod | 18/174 (10.3%) | at 6M, 1Y, 2Y, 3Y:  (estimates)  95%, 88%, 82%, 62% |
| Patil et al. [7]  (2014) | HM II (58)  HW (35) | (median) 527 | ≤ triv | ≥ mod | 13/93 (14.0%) | at 1Y, 2Y, 3Y, 4Y:  94.7%, 86.9%, 82.8%, 31% |
| Hiraoka et al. [8]  (2015) | HM II (69)  HW (11)  Ventra (2) | (mean) 314 | ≤ trace | ≥ mild | 43/82 (52.4%) | at 1Y:  35.9% |
| Imamura et al. [9]  (2015) | HM II (11)  Jarvik (3)  EvaH (24)  DuraH (14) | n/a | <mild | ≥ mild | 11/52 (21.2%) | n/a |
| da Rocha e Silva et al. [10]  (2016) | HM II (25)  HW-Lv (77) | (mean) 572 | ≤ mild | ≥ mild-mod | 32/102 (31.4%) | at 1Y, 2Y, 3Y:  (estimates)  HM: 60%, 55%, 45%  HW: 75%, 60%, 60% |
| Saeed et al. [14]  (2016) | HW-Lv (34) | (median) 408 | ≤ mild | ≥ mod | 1/34 (2.9%) | at 1Y, 2Y, 3Y:  (estimates)  100%, 100%, 88% |
| Bhagra et al. [14]  (2016) | HW-Lv (73) | (mean) 624 | ≤ mild | ≥ mod | 1/73 (1.4%) | at 1Y:  98.1% |
| Holley et al. [12]  (2017) | HM II (210) | (median) 582 | ≤ mild | ≥ mod | 32/210 (15.2%) | at 1Y, 2Y, 3Y, 4Y, 5Y:  94%, 81%, 76%, 70%, 65% |

(Abbreviations in alphabetical order). AI, aortic valve insufficiency; cf-LVAD, continuous-flow left ventricular assist device; DuraH, DuraHeart; EvaH, EVAHEART; HM II, HeartMate II; HW, HeartWare HVAD; HW-Lv, HeartWare HVAD with Lavare cycle enabled; Jarvik, Jarvik 2000; M, months; mod, moderate; Nipro, Nipro (formerly Toyobo); Nova, Novacor; n/a, data not available; pf-LVAD, pulsatile-flow left ventricular assist device; triv, trivial; Ventra, Ventrassist; XVE, HeartMate XVE; Y, year(s).
